# Automation of a fixed-bed continuous–flow reactor

**DOI:** 10.1155/S1463924694000234

**Published:** 1994

**Authors:** R. Alcántara, L. Canoira, R. Conde, J. M. Fernández-Sánchez, A. Navarro

**Affiliations:** 1 Departrnento de Ingenieria Q.uirnica y Combustibles Escuela Ténica Superior de Ingemeros de Minas Universidad Politécnica de Madrid Rios Rosas 21 Madrid 28003 Spain; 2 Instituto de Estructura de la Materia CSIC Serrano 123 Madrid 28006 Spain

## Abstract

This paper describes the design and operation of a laboratory plant with a fixed-bed continuous-flow reactor, fully automated and controlled from a personal computer. The automated variables include two gas flows, one liquid flow, six temperatures, two pressures, one circulation of a cooling liquid, and 10 electrovalves. An adaptive-predictive control system was used. The chemical process chosen to run the automated reactor was the conversion of methanol to gasoline over a ZSM-5 catalyst. This is a highly exothermal process, so a cascade control system had to be used to control the reactor internal temperature. Pressure and weight hourly space velocity (WHSV) were fixed at 1 arm and 1.5h^-1^ respectively. Accurate control (±0.2^°^C) of the reactor’s internal temperature was achieved and repeatability for the conversion of methanol to gasoline was good.


Journal of Automatic Chemistry, Vol. 16, No. 5 (September–October 1994), pp. 187–193

### Automation of a fixed-bed continuous-flow reactor

**R. Alcántara, L. Canoira*, R Conde, J. M. Fernández-Sánchez† and A. Navarro**

*Departmento de Ingeniería Química y Combustibles, Escuela Técnica Superior de Ingeneros de Minas, Universidad Politécnica de Madrid, Rios Rosas 21, 28003 Madrid, Spain*

*This paper describes the design and operation of a laboratory plant with a fixed-bed continuous-flow reactor, fully automated and controlled from a personal computer. The automated variables include two gas flows, one liquid flow, six temperatures, two pressures, one circulation of a cooling liquid, and 10 electrovalves. An adaptive-predictive control system was used. The chemical process chosen to run the automated reactor was the conversion of methanol to gasoline over a ZSM-5 catalyst. This is a highly exothermal process, so a cascade control system had to be used to control the reactor internal temperature. Pressure and weight hourly space velocity (WHSV) were fixed at 1 atm and $1{\cdot}5\,h^{-1}$ respectively. Accurate control ($\pm 0{\cdot}2°C$) of the reactor's internal temperature was achieved and repeatability for the conversion of methanol to gasoline was good.*

### Introduction

University students have few opportunities to work in the laboratories with highly automated plants, despite the importance of them in the chemical industry. With the work described in this paper, we attempted to provide students with this experience. It is vital that students see and handle the kind of instrumentation and electronics currently used in industrial environments; by doing so, they become familiar with the difficulties related to the simultaneous automatic control of several variables (temperatures, flows, pressures etc.) that can interfere with each other.

With regard to automatic control systems, the most extended approach is 'negative feedback', in particular the PID type. However, this methodology has an inherent stability problem [1] which makes it unsuitable for systems with large perturbations and/or pure delays. So a compact and stable Adaptive-Predictive Control System (APCS) [2] was used to control the automated plant described here. The APCS was run on a personal computer, so there were benefits from computer-based control: for example data storing to display time evolution, simultaneous control of a large number of variables, time schedule and synchronization of actions.

The chemical process chosen to test the automated fixed-bed continuous-flow reactor is the conversion of methanol to gasoline (MTG) over a ZSM-5 catalyst [3]:

$$2CH_3OH \leftrightarrow CH_3OCH_3 \rightarrow CH_2{=}CH_2 \rightarrow \text{gasoline} + H_2O$$

* Correspondence to Dr Canoira.

† Present address: Instituto de Estructura de la Materia, CSIC, Serrano 123, 28006 Madrid, Spain.

This process has a number of interesting features: it is the application of a chemical reaction to the production of a conventional fuel (gasoline) and it is a highly exothermal process, which makes the control of the reactor's internal temperature difficult.

### Description of the system

### *Laboratory plant*

A flow diagram of the plant is shown in figure 1, where a label is assigned to each item of equipment for identification purposes. The fixed-bed reactor (1) is a stainless steel tube, 50 cm long and 2·5 cm external diameter. This reactor is surrounded by a cylindrical heating jacket, divided into three heating zones. Each zone has its own electric resistance (R1 = R2 = 180 Ω and R3 = 129 Ω) and thermocouple to measure the three temperatures (T1, T2 and T3). The reactor internal temperature (TIR) is also measured with a thermocouple, in a shield, immersed in the catalytic bed.

Upstream from the reactor, the vaporizer (2) is a cylindrical stainless steel recipient, 10 cm long and 5 cm external diameter, filled with glass Raschig rings, and surrounded by a heating jacket. The vaporizer has two top inlets for liquid and gas feeding, and a bottom outlet, and it is equipped with a shield for a thermocouple to measure the vaporizer temperature (TEV). The gases are fed into the vaporizer from pressure cylinders through mass flow controllers FC1 (nitrogen) and FC2 (air), and liquid methanol is fed by a syringe pump (MET).

Downstream from the reactor, there is a heat exchanger (3) to cool the reaction products. It is a stainless steel cylinder, 20 cm long and 5 cm external diameter, with a top inlet and a bottom outlet. A membrane pump (BAL) makes a cooling liquid (ethylenglycol–water mixture) circulate through the heat exchanger and through a freezing bath.

The mixture of gas and liquid products goes to a gas-liquid separator (4). It is a cylindrical stainless steel recipient, 15·5 cm long and 5 cm external diameter, with one inlet and two outlets, one at the top for gas products and one at the bottom for liquid products. Liquids are emptied at a fixed time rate into a storing pot (5) and gases pass through a back-pressure regulator (P1) and a gas flowmeter (not in figure 1).

There is also a thermocouple to measure the temperature (TOUT) of the outgoing gases, and a pressure gauge (P2) to have an independent measure of the plant pressure.

Several electrovalves (V1 to V8) are placed over the plant to control the passage of flow. There are three security valves at the reactor outlet: one electrovalve (V8), one

0142-0453/94 $10.00 © 1994 Taylor & Francis Ltd.

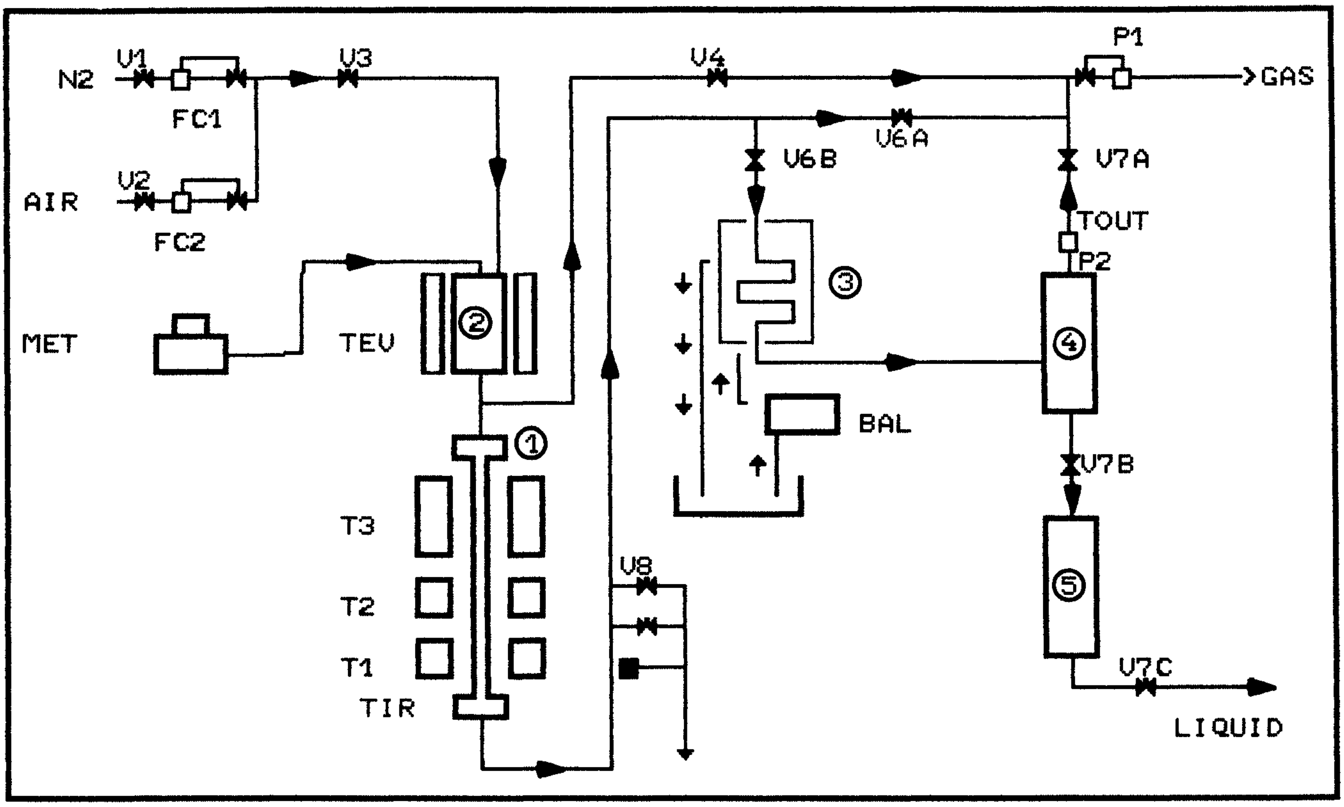

*Figure 1. Flow diagram of the automated laboratory plant.*

pressure-relief valve, and one manual valve. All the connections between these items of equipment are made in $\frac{1}{4}$ in stainless steel tube (except liquid feeding, $\frac{1}{16}$ in), using stainless steel standard fittings.

The variables which have been automated in this plant are:

(1) Gas flows (air and nitrogen).
(2) Liquid flow (methanol).
(3) Vaporizer internal temperature.
(4) Reactor internal temperature.
(5) Temperature of the three heating zones of the reactor jacket.
(6) Exit gas temperature.
(7) Pressure of the system.
(8) Circulation of the refrigerating mixture through the heat exchanger.
(9) Ten electrovalves.

An important feature of this reactor system is its versatility, since it can be used for a large variety of chemical processes, and a higher number of variables can be automated if needed.

*Transducers, actuators, and communications*

This section describes the link between the laboratory plant and the personal computer (PC).

*Transducers*

For temperature measurement K type thermocouples were used. The back-pressure regulator P1 was BROOKS 5866, with a range of 5 bar. For the pressure measure P2, a pressure transducer BOURDON XM 2000, with a 4–20 mA signal and a range of 10 bar was used. Two mass flow controllers BROOKS 5850 TR, calibrated for use with nitrogen (0–6 $l_n\,h^{-1}$) and air (0–12 $l_n\,h^{-1}$), respectively, were also used.

*Actuators*

All the electrovalves in the plant were in stainless steel from HIRSCHMANN, model 12BGPM. The cooling liquid is circulated through the heat exchanger by a membrane type double-body pump from ELECTRO AD, S.L. The syringe pump was SAGE 341B from ORION. Finally, there are four solid state relays connected in series with the main (220 VAC/50 Hz) and the electrical resistances of the vaporizer and the three heating zones of the reactor jacket.

*Communications*

The communication input/output station contains the brain boards, the communication racks and modules, and the VDC power supplies. The OPTOMUX B1 and B2 brain boards from OPTO 22 were used—these have a microprocessor and contain the intelligent circuits to communicate via serial the input/output station and the CPU of the PC. The communication protocol is RS-422/485. The boards can operate at speeds ranging from 300 to 38·4 kbauds.

Two types of racks were employed: analogue rack OPTO PB16AH, and digital rack OPTO PB16H, which can

*Table 1. Main characteristics of OPTO22 input/output modules used in the automated plant.*

| Specifications | | | |
|---|---|---|---|
| Analogue modules | Input range | Output range | Accuracy |
| AD3 | 4–20 mA | — | ±0·02 mA (0·1%) |
| AD6 | 0–5 V | — | ±0·1 mV (0·1%) |
| AD8, AD8T | −125 to 1250°C | — | ±3°C |
| DA4 | — | 0–5 V | ±5 mV (0·1%) |
| DA5 | — | 0–10 V | ±10 mV (0·1%) |
| Specifications | | | |
| Digital modules | Current rating | Turn-on/off time | Isolation |
| OAC5A | 3A | 10 ms (maximum) | 4000 $V_{rms}$ |
| IAC5A | — | 20 ms | 4000 $V_{rms}$ |

contain 16 modules each. For conditioning the electric signals from/to the plant, conversion modules from OPTO 22 were used, which are optically isolated. The modules employed and their characteristics are summarized in table 1. Finally, the VDC power supplies provide the current necessary to operate the station and the DC operated actuators.

*Computer*

The PC was an AST Premium 286 at 10 MHz, with a hard disk of 20 Mb and a 5¼″ floppy disk drive. The communication board was an MPS/88 developed by SCAP Europe S.A., based in a 16 bit 8096 microprocessor by INTEL. The PC was equipped with the following peripherals:

(1) Two monitors, a high resolution colour display for graphics, and a black-and-white monitor for text and dialogue; such a configuration allows the operator to watch, in the colour monitor, the evolution of process variables and control loops, and, in the black-and-white monitor, to modify some parameters of the process.
(2) A graphic tablet KURTA IS/ONE, to introduce logic programming and icons for graphics.
(3) A dot matrix printer.

The PC was powered through an uninterrupted power supply, that protected the computer from failures in the power lasting more than 15 min.

The QNX operating system for IBM PC by Quantum Software Systems was used, and the APCS software package, by SCAP Europe S.A. The multitask QNX operating system has an excellent capability for real time processing, so it is very appropriate for data acquisition and control applications.

The APCS software allows, in a former configuration mode, to type the APCS parameters (see below) for each control loop, to write managing programs, to store the variables, etc. In a later operation mode, it controls the defined loops, makes decisions based on the managing programs and logic programming, and allows the operator to see, in analogue (graphic) and numerical form, real time information about control loops, time evolution of process variables, etc. The operator can also change some parameters of the process, as well as to program a time schedule for the process.

*The adaptive-predictive control system (APCS)*

The main aim of any automatic control system is to keep a number of physical or chemical variables (for example pressure, temperature and pH), related to the operation of an industrial process, as close as possible to certain values previously set (setpoints), independently of the variations that can occur in the process. In any process to be controlled, there is always a process input (or control signal), that can be changed, and a process output (a measured variable), which is controlled.

The operation of the APCS has been previously described [2]. The block diagram of the APCS is shown in figure 2. The driver block calculates a 'desired process path', that the process output variable should follow to reach the setpoint, and, therefore, calculates the desired output for the next control period. The control signal (process input) is then calculated by the predictive model from the desired output, so that the predicted process output matches the desired output. The predictive model is a mathematical representation of the process: a set of mathematical equations that relate input and output variables of the process [2]:

$$\begin{aligned} \mathrm{PV}(K+1) &= A_1\mathrm{PV}(K) + A_2\mathrm{PV}(K-1) + \cdots \\ &\quad + B_1\,\mathrm{OUT}\,(K) + B_2\,\mathrm{OUT}\,(K-1) \\ &\quad + B_3\,\mathrm{OUT}\,(K-2) + \cdots \\ &\quad + C_1\,\mathrm{PERT}\,(K) + C_2\,\mathrm{PERT}\,(K-1) + \cdots \end{aligned}$$

This equation gives $\mathrm{PV}(K+1)$, the process output variable in the time interval $K+1$, as a function of those of current ($K$) and previous ($K-1, K-2, \ldots$) periods, of the control signal (OUT), and of any measurable perturbation (PERT) that can affect the process. The

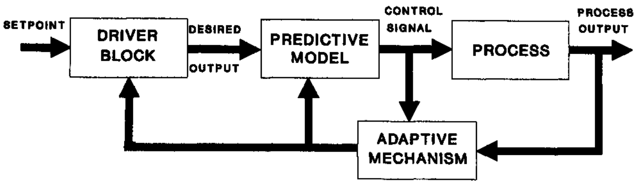

*Figure 2. Block diagram of the adaptive-predictive control system.*

parameters $A_1, A_2, \ldots, B_1, B_2, \ldots$, and $C_1, C_2, \ldots$ of the APCS model have to be typed by the operator in the configuration stage, as well as other structural parameters like control period, predictive horizon, time constant and number of pure delays.

The adaptive mechanism works, from the process inputs and outputs, at two levels:

(1) It adapts the parameters of the predictive model.
(2) It tells the driver block the evolution of the process output variable in order for the driver block to re-direct properly the desired path.

### Experimental

### *Zeolite catalyst*

ZSM-5 zeolite was synthesized in a 1 l, stainless steel Burton Coblin autoclave from Autoclave Engineers Inc., according to the hydrothermal procedure described by Costa *et al.* [4]. The resulting solid was characterized by X-ray fluorescence (chemical analysis), X-ray diffraction (crystallinity), and scanning electron microscopy (crystal morphology). A pure enough zeolite (70% crystallinity, by comparison with a reference standard) was obtained.

The catalyst for the MTG process was prepared as follows [5]. Raw ZSM-5 was exchanged with 0·6 M HCl to produce the acid form HZSM-5, and then bound with 30% w/w sodium montmorillonite clay, kindly supplied by Minas de Gador, S.A. The fraction between 0·5 mm and 1 mm particle size (18·62 g of catalyst) was used as catalyst, which occupied approximately the two bottom zones (8 cm length) of the reactor tube.

### *Operation*

The vaporizer temperature was set at 200°C for all experiments. Before each experiment, the catalyst was activated at 450°C for 4 h in nitrogen flow. After this, the reactor was allowed to cool to the optimal reaction temperature [3] (370°C), under the same nitrogen flow, and then the methanol feeding pump was switched on automatically. All the experiments were carried out at atmospheric pressure, and with a small nitrogen flow (0·61 $l_n$ $h^{-1}$) to favour the transport of methanol vapour. The plant is operated at a weight hourly space velocity (WHSV) of 1 $h^{-1}$, since this space velocity has proved to be an optimal value for the methanol to hydrocarbons conversion over ZSM-5 catalyst [3].

After finishing each experiment, a nitrogen flow was used to desorb the products that remain in the catalyst, while the temperature was kept at 370°C. Regeneration of the catalyst was carried out at 550°C in air flow for 3–4 h.

Analytical grade methanol PANREAC was used as starting reagent. In a typical 1 h experiment some 25 g of liquid products were obtained. The liquid products were separated in a decantation funnel, aqueous and organic layers were weighed separately, and the organic fraction was analysed by gas chromatography (GC). Representative samples of gas products were also analysed by GC.

The GC analyses were performed in a Hewlett-Packard 5840 GC instrument, equipped with two FID detectors and a gas sampling valve. The analysis of liquid products was carried out in a stainless steel column (50 cm length, $\frac{1}{8}$ in o.d.), packed with UCW 982 as stationary phase and Chromosorb WAW DMCS as solid support. The analysis of gaseous products was carried out in a fused silica semicapillary column (50 cm length, 0·53 mm o.d.) of Alumina KCl PLOT from CHROMPACK. In these analyses, only hydrocarbons in the range $C_1$–$C_{10}$ were detected, in addition to unreacted methanol. The hydrocarbon products were identified and quantified by comparison with standards from Alltech Associates (liquids) and Sociedad Española del Oxigeno (gases), using the internal normalization technique.

A conversion of methanol to gasoline of around 98% was attained. The repeatability of the reactor for the conversion of methanol is good (in all experiments, it was higher than 97%.)

### Results and discussion

### *Automation of the plant*

In addition to an accurate control of the automatically controlled loops, the aim was to achieve a complete automation of the plant, so that the operator only had to start the system and, eventually, change the setpoints if needed. That automation can be obtained by means of sequentially programmed tasks, and decision-making units able to supervise the correct working of the process. The automation of the plant is described now in some detail.

First, for the computer to know the input variables and send output variables, a software variable had to be assigned to each i/o module in the communication station. Some of the input variables acted as process variables of control loops (for example temperatures TEV and TIR), and some others were just informative variables (for example TOUT and P2); among the output variables, some of them (for example voltage to the relays R1, R2 and REV) acted as control signals of control loops, and others (like those associated to the electrovalves) were output signals not involved in any control loop.

For some of the i/o variables, the electric signal was not proportional to the physical variable in the plant, so that a 'linearization function' had to be determined. This was specially important for the solid state relays. The response curve of the relay R1 of the reactor heating jacket is shown in figure 3. That curve, conveniently sampled at 13

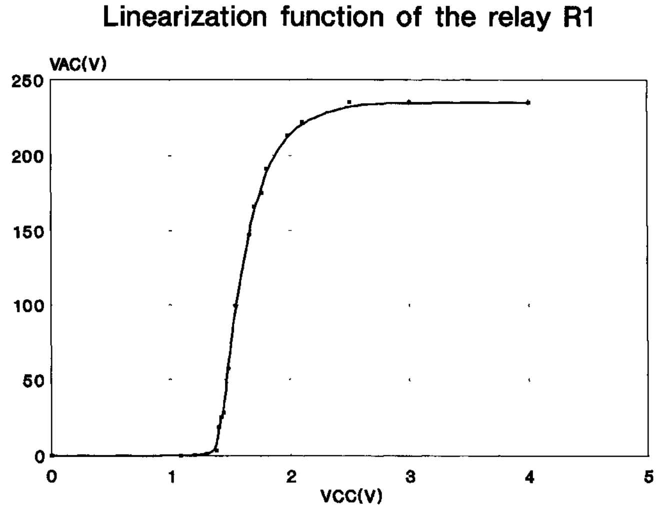

*Figure 3. Linearization function of the relay R1 of the reactor heating jacket. The ordinate axis represents the voltage drop in the resistance of the reactor heating jacket, and the abscissa axis respresents the voltage output of the DA4 OPTO22 module.*

**MANAGING PROGRAM**

PG # 32 ASSOCIATED LOOP GENERAL DESCRIPTION REACTION
EXECUTION TYPE P PERIOD 20 * 0.2 s = 4 s

| | CONDITION | | | | ACTION | | |
|---|---|---|---|---|---|---|---|
| # | TYPE | ARGUMENT 1 | OPER | ARGUMENT 2 | ARGUMENT1 | OP | ARGUMENT2 |
| 1 | EJ | | | | SP[T1] | = | 335 |
| 2 | | | | | SP[T2] | = | 335 |
| 3 | | | | | SP[T3] | = | 150 |
| 4 | | | | | SP[TEV] | = | 200 |
| 5 | | | | | V1 | = | 1 |
| 6 | | | | | V2 | = | 0 |
| 7 | | | | | V2A | = | 0 |
| 8 | | | | | V3 | = | 1 |
| 9 | | | | | V4 | = | 0 |
| 10 | | | | | V6A | = | 0 |
| 11 | | | | | V6B | = | 1 |
| 12 | | | | | V7A | = | 1 |
| 13 | | | | | V7B | = | 0 |
| 14 | | | | | V7C | = | 0 |
| 15 | | | | | MA[FC1] | = | 5 |
| 16 | | | | | PV[TIME] | = | 0 |
| 17 | RS | PV[TIR] | > | 340 | | | |
| 18 | O | PV[TIR] | < | 330 | | | |
| 19 | EJ | | | | MA[FC1] | = | 1.25 |
| 20 | | | | | BAL | = | 1 |
| 21 | | | | | MET | = | 1 |
| 22 | | | | | CPG | = | 18 |
| 23 | RS | PV[TIME] | < | 60 | PV[TIME] | + | 1 |
| 24 | EJ | | | | MA[FC1] | = | 0.6 |
| 25 | | | | | MO[TIR] | = | AUTO |
| 26 | | | | | SP[TIR] | = | 370 |
| 27 | | | | | CPG | = | 15 |
| 28 | RS | PV[TIME] | < | PV[TI_REAC] | PV[TIME] | + | 1 |
| 29 | EJ | | | | PV[FINISH] | = | 1 |

*Figure 4. Program REAC for automatic operation of the plant in the reaction mode.*

discrete points, was typed into the computer, and the same was done for all the other relays.

For the repetitive tasks, several managing programs, REGE, ACTI, and REAC were written for regeneration, activation, and reaction, respectively. As an example, the reaction program is shown in figure 4. In this program the setpoints of the reactor heating jacket are fixed at 335°C (T1 and T2), and 150°C (T3), the vaporizer is heated at 200°C and the nitrogen flow is lowered. When the reactor internal temperature (TIR) reaches 330°C, the next sentence switches on the methanol syringe pump and fixes the methanol flow. After the time fixed for the

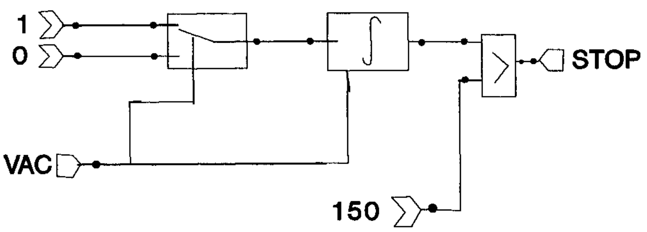

*Figure 5. Logic program block to check for the AC main.*

reaction, the REAC program stops and the REGE program starts.

These programs are easy to write and no programming skills are needed; they are chained in the sequence ACTI-REAC-REGE, so that at the end of each program, the next one starts automatically. On top of this sequence, there is a DIRECTOR program that acts as system supervisor: it can stop the system at any time if an alarm condition occurs, or if a special key is entered by the operator.

The set of managing programs included also a DISCHARGE program that dumps the liquid products into the pot at a fixed time rate, and a SAFETY program that opens the electrovalve V8 if the system pressure is raised to 5 atm.

The system also uses logic programming to make decisions. In figure 5, a safety logic block is shown that continuously checks for AC main (VAC = 1). If a failure occurs (VAC = 0), a counter starts. The system waits for 10 min for the power to return, and, after that time, all heatings and flows are set to zero, all valves are closed, and everything is stopped.

To monitor the process in real time, a synoptic screen of the plant was created. In this screen, shown in figure 1, process variables were displayed and refreshed in real time, and digital variables (like those associated to electrovalves) were shown using a colour code (for example red = closed, green = open). To allow the time evolution of the process to be displayed, the values of the temperature loops were stored every 6 s over three days.

The temperature loops TEV, T1, T2, and T3 were first tuned with a 'dead' reactor (no chemical reaction present). From the response to a step control signal, the response time, the gain and the number of delays of the loop could be determined. The control period for each loop is related to the response time of the process: it has to be large enough not to oscillate, but not so large that the control loop becomes too slow. For the temperature loops, 10 s for TEV and 7 s for T1, T2, and T3 were used. The behaviour of T3 loop is shown in figure 6. Ninety per cent of the setpoint value is reached within 15 min, and after 30 min a fine stabilization of ± 1°C is achieved.

When reactants were fed, the main problem in operating the plant was the stabilization of the reactor internal temperature at the set value, due to the high exothermicity of the MTG process.

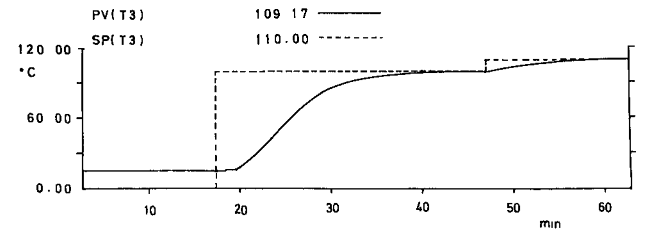

*Figure 6. Time evolution of the process variable (PV) and setpoint (SP) of the temperature T3 of the reactor heating jacket.*

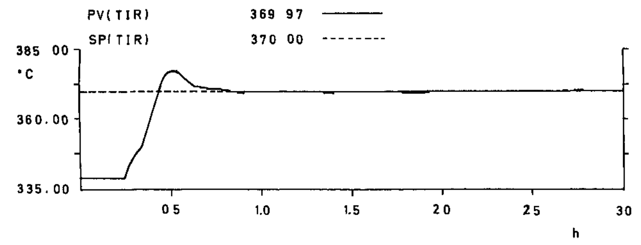

*Figure 8. Time evolution of the process variable (PV) and setpoint (SP) of the TIR control loop.*

*Control of the reactor internal temperature*

Special attention was paid to the control of the reactor internal temperature (TIR), because it is crucial for the satisfactory operation of the plant. It proved to be difficult to control due to the high reaction enthalpy of $-1250$ to $-1674$ kJ/kg and to a delay of around 2 min in the reactor heating system. The reactor had a heating system, but no cooling system, so the only way to cool it was to stop heating and to allow the heat to dissipate. This is a slow process, due to the insulating materials of the oven (asbestos and and glass wool). For these reasons, a cascade control system had to be used to get a satisfactory control of the TIR loop.

The cascade control is shown in figure 7. The control signal of the master loop is used as setpoint for the slave loop(s). The response time of the slave loop(s) must be much shorter than that of the master loop for the cascade control system to work properly. This condition is fulfilled by the TIR as the master loop and the T1 and T2 temperatures of the zones 1 and 2 of the heating jacket as the slave loops. The optimal control period for the master loop was 1·2 min and those of the slave loops were 7 s. Zone 3 of the reactor heating jacket was excluded from the cascade control system, because the catalyst only occupied the two lower zones of the reactor.

The start-up of the plant posed another problem. When feeding methanol, the TIR rapidly increased 30–40°C, due to the reaction heat. To account for this effect, TIR was set to 340°C, 30°C less than the optimal 370°C, before starting feeding methanol.

Figure 8 shows the process variable (PV) and the setpoint (SP) of the TIR control loop vs time. TIR was at 340°C for 15 min, and after starting feeding methanol there is a rapid rise in temperature to almost 380°C, but in less than 10 min the TIR is stabilized to 370°C, where it is held.

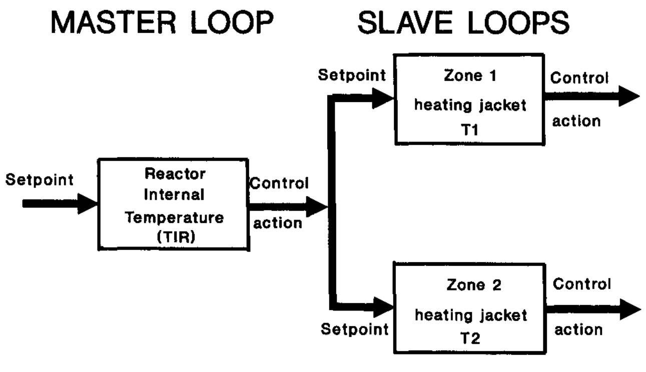

*Figure 7. The cascade control system.*

The activity of the catalyst, measured by the production of heat within the reactor, can be followed by means of the temperature difference between the reactor interior (TIR) and the reactor jacket (T1 and T2). This activity was decreasing to some extent during the experiment, but the control system held the reactor internal temperature within $\pm 0{\cdot}2\%$ of the set value.

### Concluding remarks

It has been demonstrated that a continuous-flow laboratory plant can be automated using available technology and electronics, and an adaptive-predictive control software. Among all the control loops, the most difficult was the reactor internal temperature, due to the highly exothermal reaction and heating delays. For a satisfactory control of this variable, a cascade control system was chosen. With this system, a fine control of $\pm 0{\cdot}2$°C of the reactor internal temperature was achieved.

This work also shows the application of the novel approach of adaptive-predictive control to a chemically interesting process. The methodology has the advantages of computer-based control, like capability of data storing and evolution, simultaneous control of interrelated variables, and of a stable control system. The main drawback of the system would be its rather complex configuration (tuning) stage (especially for people who are more familiar with tuning PID type controllers), and its price.

It is hoped that this paper will encourage the application of advanced control techniques and technology to the automation of plants and pilot models in university laboratories.

### Acknowledgements

We are very grateful to the company SCAP EUROPE S.A. for its control software package, and to the company's engineers J. M. Martin Sánchez, Leopoldo Pérez and Javier Cerezo for their technical support. We are also indebted to the Laboratorio Centralizado of the E.T.S.I. Minas for characterization analyses of the zeolite samples.

### References

1. Gómez Iniesta, A., de Pablo Olaiz, J. C. and Rodellar Benedé, J., *Mundo Electrónico*, **198** (1989), 71–78.
2. (a) Martín-Sánchez, J. M., *Proceedings of the Institute of Electrical and Electronics Engineers*, **64**(8) (1976), 1209–1218; (b) Martín-Sánchez, J. M., U.S. Patent 4,197,576 (1980); (c) Martín-Sánchez, J. M. and Shah, S. L., *Automatica*, **20**(5) (1984), 607–620.
3. Chang, C. D., *Catalysis Reviews—Science and Engineering*, **25**(1) (1983), 1–118.
4. Costa, E., Uguina, M. A., de Lucas, A. and Blanes, J., *Journal of Catalysis*, **107** (1987), 317–324.
5. Costa, E., Aguado, J., Uguina, M. A. and Fernandez de Castro, A., *Revista Latinoamericana de Ingeniería Química Aplicada*, **16** (1986), 87–100.

